# RUNX1 recruitment of GCN5 in keratinocytes upregulates ICOSLG and promotes T cell activation in the psoriasis microenvironment

**DOI:** 10.1038/s12276-026-01738-8

**Published:** 2026-06-04

**Authors:** Lifei Zhu, Wen Wang, Jieyu Lin, Lu Peng, Zhu Shen

**Affiliations:** 1 Department of Dermatology, Guangdong Provincial People’s Hospital, Guangdong Academy of Medical Sciences, Southern Medical University, Guangzhou, China; 2 Department of Oncology, The fifth Affiliated Hospital of Sun Yat-sen University, Zhuhai, China

**Keywords:** Psoriasis, Immunoproliferative disorders, Acetylation

## Abstract

Psoriasis is an immune-mediated disorder whose recurrence is primarily driven by cutaneous-resident T cells. Cutaneous T cells produce high levels of pathogenic cytokines, which subsequently initiate and sustain hyperproliferation and aberrant inflammatory cascades in keratinocytes. However, how keratinocytes orchestrate the activities of the cutaneous T cells remains elusive. Herein, single-cell RNA-sequencing and multiplex immunohistochemistry staining revealed that psoriatic keratinocytes upregulated the expression of T cell co-stimulator inducible T cell co-stimulator ligand (ICOSLG) and RUNX1, which showed a positive correlation. Multiplex immunohistochemistry further proved that ICOSLG colocalized with ICOS on T cell surfaces within psoriasis lesions, indicating that the ICOSLG–ICOS signal mediated keratinocyte-dependent T cell activation in psoriatic inflammation. Mechanistic investigations showed that RUNX1 transcriptionally upregulated ICOSLG expression via recruiting GCN5 (general control non-derepressible 5) to ICOSLG promoter, and the GCN5 inhibitor butyrolactone 3 (MB-3) reduced ICOSLG expression in keratinocytes. In a mouse model of psoriasis, MB-3 treatment ameliorated imiquimod-induced psoriatic lesion and its recurrence by suppressing keratinocyte ICOSLG expression and subsequent T cell activation. Notably, our keratinocyte-specific GCN5 knockdown mouse model demonstrated that this genetic ablation impaired the therapeutic benefits of MB-3. Taken together, these findings uncover the molecular mechanism driving ICOSLG upregulation in keratinocytes. Our study supports that clinical evaluation of the GCN5 inhibitor MB-3 is warranted for patients with psoriasis with ICOSLG overexpression.

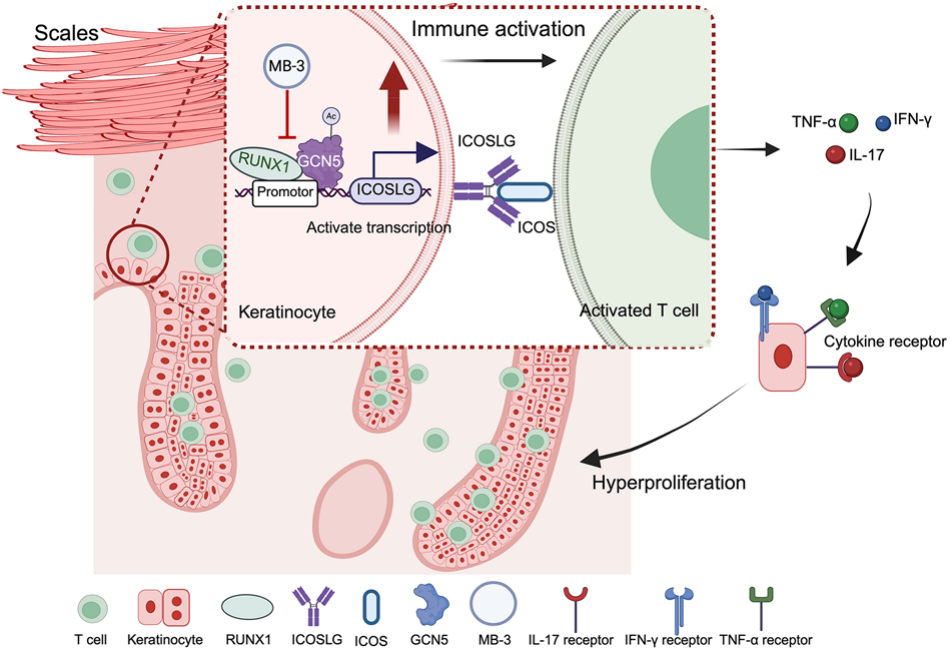

## Keratinocyte–T cell activation drives psoriasis inflammation

Psoriasis, a chronic inflammatory skin disorder, involves complex interactions between keratinocytes and T cells. This study explores the role of the inducible T cell co-stimulator ligand (ICOSLG) in psoriasis, revealing its upregulation in psoriatic keratinocytes and its correlation with disease severity. Using single-cell RNA-sequencing and immunohistochemistry, the authors identified a positive correlation between ICOSLG and RUNX1 expression, suggesting a mechanism in which RUNX1 recruits GCN5 (general control non-derepressible 5) to enhance ICOSLG transcription. The study demonstrated that inhibiting GCN5 with MB-3 reduced ICOSLG expression, alleviating psoriasis symptoms in a mouse model. These findings highlight the potential of targeting the RUNX1–GCN5–ICOSLG axis as a therapeutic strategy for psoriasis, offering a new avenue for treatment development. Future research could further explore the therapeutic potential of GCN5 inhibitors in managing psoriasis and preventing disease recurrence. IFN-γ, interferon-gamma; TNF-α, tumor necrosis factor-alpha. This summary was initially drafted using artificial intelligence, then revised and fact-checked by the author.

## Introduction

Psoriasis is a chronic immune-mediated inflammatory disorder characterized by epidermal hyperplasia and immune cell infiltration, with cutaneous-resident T cells emerging as key drivers of disease recurrence^1–4^. Keratinocytes, vital to psoriasis pathogenesis, contribute to disease initiation and maintenance by modulating gene expression to recruit, sustain, and activate local T cells^5,6^. The interactions between keratinocytes and T cells within the epidermal microenvironment have long been a focal point of research.

Over the past two decades, significant progress has been made in this field. For instance, IL-22, secreted by activated T cells, acts as a potent inducer of keratinocyte proliferation during inflammation. Keratinocytes, in turn, produce cytokines such as tumor necrosis factor-alpha (TNF-α), IL-17, IL-23, IL-6, and CCL27, which potently recruit T cells^7,8^. Concurrently, IL-23 mediates the pro-inflammatory functions of T_H_17 cells by promoting their differentiation and sustaining the production of inflammatory cytokines, including IL-17A, IL-22, and TNF-α^9^. These processes collectively establish a nascent positive feedback loop within the immune microenvironment of psoriatic lesions. However, the direct interplay between keratinocytes and T cells remains incompletely understood, with additional regulatory factors yet to be identified.

The inducible T cell co-stimulator (ICOS), an activating receptor expressed on the surface of activated T cells, has been implicated in T cell biology. Mackay and co-workers identified the ICOS–c-Maf–IL-7 axis as critical for the generation and differentiation of skin-resident IL-17-producing T cells^10^. ICOSLG (ICOS ligand), which is highly expressed on the surface of many antigen-presenting cells (APCs)^11^, activates the ICOSLG–ICOS signaling axis to support the development and function of resident T cells^12,13^, suggesting a potential role for ICOSLG in the psoriatic immune microenvironment. Nevertheless, the mechanisms underlying ICOSLG regulation in psoriasis remain unclear.

In our study, single-cell RNA-sequencing (scRNA-seq) and multiplex immunohistochemistry (mIHC) analyses demonstrated upregulated expression of ICOSLG and RUNX1 in psoriatic keratinocytes; furthermore, ICOSLG expression was positively correlated with both RUNX1 expression levels and the clinical disease severity as measured by the Psoriasis Area and Severity Index (PASI) score. mIHC further demonstrated colocalization of ICOSLG with ICOS on T cells within psoriatic lesions. Mechanistically, RUNX1 upregulated ICOSLG by recruiting GCN5 (general control non-derepressible 5) to the ICOSLG promoter. Notably, treatment with the GCN5 inhibitor MB-3 reduced ICOSLG expression in keratinocytes and alleviated both imiquimod-induced psoriasis and its recurrence by suppressing ICOSLG and T cell activation. Collectively, these findings uncover a mechanism driving ICOSLG upregulation in keratinocytes, providing a rationale for evaluating GCN5 inhibitors in the treatment of ICOSLG-overexpressing psoriasis.

## Materials and methods

### Cell culture

Normal human epidermal keratinocytes (NHEKs), HaCaT, and human embryonic kidney (HEK)-293T cells were obtained from American Type Culture Collection. Primary T cells were isolated from human peripheral blood mononuclear cells using the Naive Pan T Cell Isolation Kit (Miltenyi Biotec, Germany). NHEKs and HaCaT were cultured in DMEM (Invitrogen, USA) supplemented with 10% fetal bovine serum (EXCELL, Suzhou, China). Primary T cells were grown in RPMI 1640 (Invitrogen) supplemented with 10% fetal bovine serum (Invitrogen), 100 IU/ml penicillin, and 100 μg/ml streptomycin (Invitrogen). All of the cells were maintained at 37 °C and 5% CO_2_ humidified atmosphere.

### Plasmid and RNA transfection

The SFB-tagged RUNX1, HA-tagged CBP, P300, PCAF, GCN5, and GFP-ICOSLG were cloned into the pSIN-puro vector, respectively. Different fragments of the ICOSLG promoter region were cloned into the pGL3-basic vector. Lipofectamine@3000 transfection reagent (Thermo Fisher, USA) was used to transient transfect the above plasmids. The small interfering RNAs (siRNAs) were constructed by Guangzhou Ribo Biotechnology Co., Ltd. The siRNAs experiments were performed using RNAiMAX reagents (Thermo Fisher) by reverse transfection of the aforementioned siRNAs according to the manufacturer’s instructions. The sequences of siRNAs were listed subsequently: siRUNX1-1 5′-GCACTCTGGTCACTGTGAT-3′, siRUNX1-2 5′-CCAGGTTGCAAGATTTAAT-3′, si-ICOSLG-1 5′-GGATCAATAAGACGG ACAA-3′, si-ICOSLG-2 5′-CGTTGAGGTTACACTGCAT-3′, si-GCN5-1 5′-TTATACCAGATGGCTCTGTTA-3′, and si-GCN5-2 5′-AGTACGCCATTGGCTAC TTCA-3′.

### Immunoblotting and immunoprecipitation

After harvesting, cells were lysed using RIPA buffer for protein extraction. The antibodies used in this study were as follows: ICOSLG (1:500, Abcam, UK), RUNX1 (1:1,000, GeneTex, USA), GCN5 (1:1,000, GeneTex), HA (1:2,000, Cell Signaling Technology, USA), GFP (1:2,000, Cell Signaling Technology), and GAPDH (1:8,000, Proteintech Group, Inc., China). For immunoprecipitation (IP), anti-Flag, anti-HA agarose beads (Sigma-Aldrich, USA) were washed three times for 5 min with RIPA buffer. Subsequently, lysates were incubated with 15 µl beads overnight at 4 °C and then washed, and the precipitates were analyzed by western blotting. For endogenous IP, protein A/G agarose beads were washed with RIPA buffer for three times. Washed agarose beads along with antibodies against GCN5, RUNX1, and control rabbit IgG were incubated with the indicated protein supernatant (500 μl) overnight at 4 °C, followed by western blotting analysis.

### RNA extraction and RT-qPCR experiment

Total RNA extraction was performed using the Omega E.Z.N.A.® Total RNA Kit I (Omega Bio-tek, USA), according to the product manual. About 1 μg mRNA was subjected to synthesize cDNA using Hifair® III 1st Strand cDNA Synthesis SuperMix for quantitative PCR (qPCR) (Vazyme, China). Then, quantitative real-time PCR experiments were carried out using Hieff® qPCR SYBR Green Master Mix (Vazyme) and Roche Light Cycler 480 instrument.

### ChIP-qPCR assay

The chromatin immunoprecipitation (ChIP) experiment was performed following the manufacturer’s protocol of the SimpleChIP® Enzymatic Chromatin IP Kit (Cell Signaling Technology). Briefly, the indicated cells were fixed by 1% formaldehyde to crosslink proteins to DNA. After sample collection, chromatin was digested with micrococcal nuclease by incubating samples on a rotator at 37 °C for 20 min, resulting in DNA fragments of ~150–900 bp long. Purified chromatin was then immunoprecipitated using RUNX1 antibody and protein G agarose beads. Following IP, the agarose beads were washed, and DNA–protein crosslinks were reversed. Finally, the purified DNA was analyzed by qPCR. The primer sequences were listed below: ICOSLG (−536 bp to 36 bp), forward 5′-AGGTACCGAGCTCTTACGC-3′, reverse 5′-CACCTGAATCCCCTCCACC-3′; ICOSLG (−995 bp to −536 bp), forward 5′-GTCTCTCAGCGTGGCCTG-3′, reverse 5′-GCTGCCATTCCTTCAACCAA-3′; ICOSLG (−1,356 bp to −995 bp), forward 5′-GTCTAGCAGCCTCACCCAG-3′, reverse 5′-CAGCTGGTTTGGGAAAGGC-3′; and ICOSLG (−2,000 bp to −1,356 bp), forward 5′-TCCTTCCATTCATGCATGCG-3′, reverse 5′-CACAATGCGCCGGTCCTG-3′.

### Dual-luciferase reporter assay

NHEK cells were seeded into a 24-well plate at a density of 1 × 10^5^ cells per well and then transfected with 0.5 μg of ICOSLG promoter luciferase plasmid, along with 10 ng of Renilla luciferase plasmid to normalize the transfection efficiency. The luciferase activity was measured by Dual-Luciferase Assay kit (Promega, USA) after 48 h transfection according to the manufacturer’s instruction.

### Co-culture and Cytokine ELISA assay

Primary T cells and HuT 78 cells were activated with 10 μg/ml CD3 and CD28 for 48 h. Subsequently, 1 × 10^4^ NHEK cells or 0.5 × 10^4^ HaCaT cells were plated in 96-well plates. After cell adhesion, primary T cells or HuT 78 cells were co-cultured with NHEK or HaCaT cells at a 4:1 ratio in 200 μl of culture medium. The supernatants or mouse serum was collected and analyzed using the Human IL-17 Quantikine ELISA Kit (R&D Systems, USA), Human TNF-α ELISA Kit (Proteintech Group, Inc., China), Human IFN-γ ELISA Kit (Proteintech Group, Inc.), Human IL-22 ELISA Kit (Coibo Bio, China), and Amplex Red ALT/AST activity assay kit (Beyotime, China), following the manufacturer’s protocol.

### Immunohistochemistry and multiplex immunohistochemistry

IHC staining was conducted on 4-μm-thick paraffin-embedded skin sections. Sections were then treated with 0.3% H_2_O_2_ in methanol for 30 min to block endogenous peroxidase activity. Primary antibodies targeting ICOSLG, RUNX1, and ki-67 were diluted at a ratio of 1:200 or 1:300, respectively, and incubated overnight at 4 °C. Finally, the sections were counterstained with hematoxylin to visualize cell nuclei. We conducted mIHC staining using the PANO 7-plex IHC kit (Panovue, China) according to manufacturer’s protocol. High-resolution whole-slide fluorescence images were acquired using an Olympus VS200 automated slide scanning system (Olympus Corporation, Japan).

### Participants and tissue samples

Psoriatic skin specimens were collected from consenting patients with psoriasis under standardized clinical conditions. This study was strictly adhered to the ethical principles outlined in the Declaration of Helsinki. All samples were collected with the participants’ written informed consent and approval from the Institutional Review Board of Guangdong Provincial People’s Hospital (Approval No. KY2024-811-01).

### Human scRNA-seq and data analysis

A single-cell suspension of skin cells was generated from three patients with psoriasis paired with three control samples. Sorted cells were subjected to single-cell 5’ library construction using the 10× Genomics platform, followed by sequencing on an Illumina instrument. Subsequent data analysis, including principal component analysis and differential expression, was performed with the Pegasus toolkit, and results were visualized using ggplot2 in R. Single-cell expression data with cell type annotations are analyzed using CellPhoneDB to identify significant ligand–receptor interactions.

### Plasmid construction and adeno-associated virus production

siRNA sequences targeting the GCN5 gene (TTATACCAGATGGCTCTGTTA) and a scrambled negative control (TTCTCCGAACGTGTCACGT) were synthesized and cloned into the GV478 vector (pAAV-k14 promoter‑EGFP‑MIR155(MCS)‑SV40 polyA). The viral vector, together with the pHelper and pRepCap plasmids, was transfected into 293T cells using Lipofectamine 2000 (Invitrogen). AAV9 serotype viruses were purified by iodixanol gradient ultracentrifugation and subsequently concentrated. Titers of purified adeno-associated virus (AAV) were determined using a quantitative PCR‑based method. All AAV preparations used in this study were formulated in 0.001% Pluronic F‑68 solution (Poloxamer 188 Solution, PFL01‑100ML, Caisson Laboratories, Smithfield, UT, USA).

### Animal study

Female BALB/c mice aged 6–8 weeks were obtained from Beijing Vital River Laboratory Animal Technology and acclimatized for 1 week before experiments. All animal care and experimental procedures adhered to National Research Council’s Guide for the Care and Use of Laboratory Animals and were approved by the Animal Care Committee of Guangdong Provincial People’s Hospital (Approval No. KY2024-1186-01). At the experimental end point, blood was collected from the mice before they were euthanized, and liver and skin tissues were then harvested for analysis.

### Cell isolation and flow cytometry analysis

For in vitro experiment, the indicated cells were dissociated into single-cell suspensions. Cells were then resuspended for staining with either PE-human α-ICOSLG (eBioscience, USA) or Mouse IgG1 kappa Isotype Control-PE (eBioscience) for 15 min on ice. After staining, the cells were washed again with cold PBS gently and analyzed using flow cytometry. For in vivo experiment, mouse psoriasiform tissues and control tissues were cut into small fragments of ~2–3 mm in size. The tissue fragments were then digested in 2–3 ml of enzyme digestion solution (RPMI 1640 containing 3.5 mg/ml collagenase II, 0.02 mg/ml DNase I, and 0.002 mg/ml hyaluronidase) at a 37 °C incubator with gentle shaking for 60 min. The dissociated cell suspension was filtered through a 70 μm cell strainer, and the cell pellet was washed twice with complete medium. Before staining, cells were incubated with an Fc receptor blocking solution (anti-mouse CD16/32, BioLegend) for 10 min. Finally, single-cell suspensions were stained with anti-PerCP-CD3 (17A2, cat. no. 100288), BV605-CD4 (RM4-5, cat. no. 100547), FITC-CD8 (53-6.7, cat. no. 100706), BV421-CD25 (PC61, cat. no. 102033), APC-TCRγδ (GL3, cat. no. 118116), and APC-CD278 (ICOS, C398.4A, cat. no. 313510) monoclonal antibodies (all from BioLegend). For T_H_17 and regulatory T (T_reg_) cells detection, suspension was stained intracellularly for anti-PE/Cyanine7-IL-17A (TC11-18H10.1, cat. no. 506922) or for PE-Foxp3 (MF-14, cat. no. 126404) monoclonal antibodies, according to the manufacturer’s instructions. Flow cytometry was performed using an LSRFortessa flow cytometer (BD Biosciences), and cell sorting was conducted with a BD FACSAria II sorter. Acquired data were analyzed with FlowJo software (version 10).

### Statistical analysis

All experimental data in this study were obtained from at least three independent replicates. Data were plotted and analyzed using GraphPad 8.0. Quantitative data are presented as mean ± standard deviation (mean ± SD). Comparisons between two independent samples were performed using Student’s *t* test or one-way analysis of variance, with a significance level of *P* < 0.05 indicating statistically significant differences.

## Results

### Upregulation of ICOSLG and RUNX1 in keratinocytes of psoriatic lesions, with ICOSLG positively correlating with psoriasis severity

**Fig. 1:**
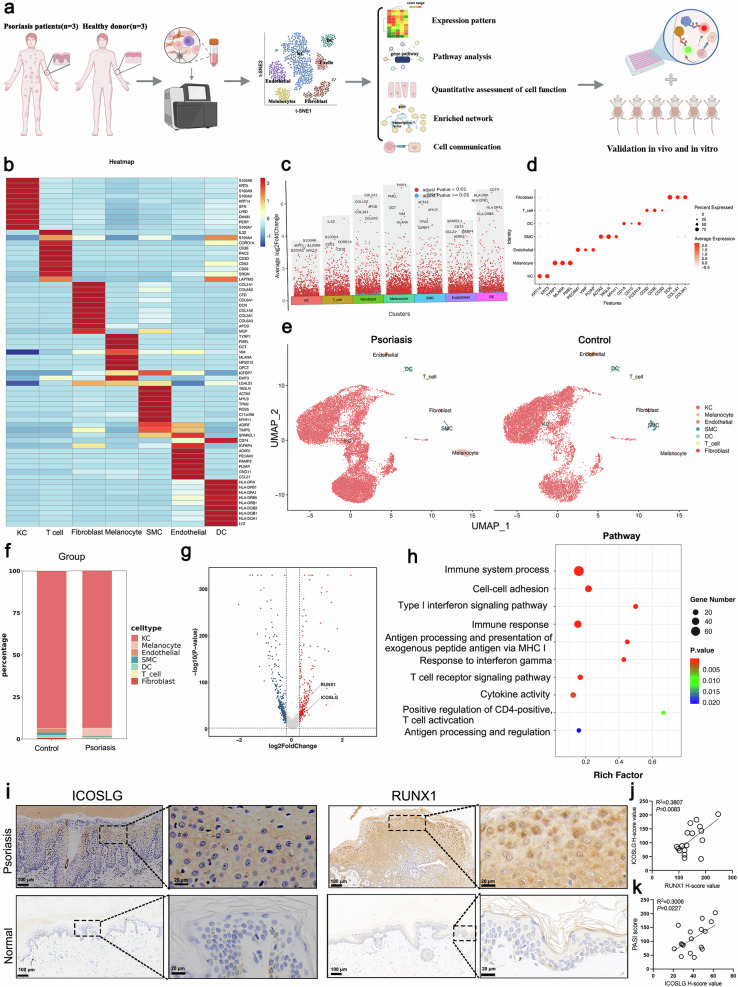
Upregulation of ICOSLG and RUNX1 in keratinocytes of psoriatic lesions. **a** Schematic diagram of the study design. **b** Heatmap and plot of the cluster-marking genes differentially expressed in each cluster. Rows represent genes, and columns represent cells grouped by cluster assignment as shown in part **e**. Color intensity indicates relative expression levels from low (blue) to high (red). **c**, **d** Transcripts significantly enriched in seven clusters from the psoriasis tissue sources. **e** Single-cell transcriptomic landscape of human skin revealed by UMAP visualization. Projection represents high-quality single cells from three patients with psoriasis and healthy controls, with colors denoting distinct cell populations identified through unsupervised clustering. **f** Proportions of cell types in the skin of the psoriasis group and healthy group. **g** Volcano plot showing differentially expressed genes in psoriatic keratinocytes by single-cell RNA-sequencing. **h** Significantly activated pathways in psoriatic keratinocytes. **i** Immunohistochemical analysis of psoriatic skin tissues paired with healthy skin tissues using anti-ICOSLG and anti-RUNX1 antibodies. Scale bars, 100 μm and 20 μm. Quantification of ICOSLG and RUNX1 expression using ImageJ. Spearman correlation analysis was performed to assess the relationship between the expression levels of ICOSLG and RUNX1 in lesional skin (part **j**) and the corresponding PASI scores (part **k**). Data were shown as mean ± SEM and were analyzed using Spearman correlation analysis. DC dendritic cell, ICOSLG inducible T cell co-stimulator ligand, KC keratinocyte, MHC major histocompatibility complex, PASI Psoriasis Area and Severity Index, SMC smooth muscle cell, t-SNE t-distributed stochastic neighbor embedding, UMAP uniform manifold approximation and projection.

To investigate the cellular and molecular mechanisms underlying the distinct patterned immune activity in psoriatic skin, we first performed scRNA-seq on skin biopsies from patients with psoriasis and healthy donors. Cell type annotations were determined using both known cellular markers and the most significantly expressed genes in each cluster (Fig. 1a–d). Following dimensionality reduction via principal component analysis and uniform manifold approximation and projection, clustering analysis of more than 50,000 single cells from three patients with psoriasis and matched healthy donors — using shared nearest neighbor optimization — yielded seven distinct clusters (Fig. 1e). For robust data comparison, compositional analysis of the single-cell transcriptomic data identified keratinocytes as the most abundant cell type (Fig. 1f), a finding consistent with the established pathophysiological mechanisms underlying psoriasis.

Next, we analyzed the gene expression patterns of keratinocytes in psoriasis, and the transcriptional profiling revealed higher expression levels of ICOSLG and RUNX1 in psoriasis keratinocytes compared with healthy donors (Fig. 1g and Supplementary Fig. 1a). Notably, transcriptomic analysis revealed significant enrichment of pathways related to antigen binding, immune response activation, interferon-gamma (IFN-γ) production, and antigen processing and presentation (Fig. 1h and Supplementary Fig. 1b–e).

To validate these scRNA-seq findings, we next conducted IHC staining using ICOSLG and RUNX1 antibody. Consistent with our observation in scRNA-seq, IHC staining demonstrated markedly increased expressions of ICOSLG and RUNX1 in psoriatic lesional skin versus healthy skin (Fig. 1i). Of note, we found that the global levels of ICOSLG were positively correlated with RUNX1 expression (*P* = 0.0083, *R*^2^ = 0.3807; Fig. 1j and Supplementary Fig. 2a). Further analysis showed a significant positive correlation between ICOSLG expression levels and patient PASI scores (*P* = 0.0227, *R*^2^ = 0.3006; Fig. 1k), whereas no statistically significant correlation was observed between RUNX1 expression levels and PASI scores (*P* = 0.1189, *R*^2^ = 0.1542; Supplementary Fig. 2b). Collectively, these results demonstrated that ICOSLG and RUNX1 were upregulated in keratinocytes of psoriatic lesion and they were positively correlated in psoriasis, which might contribute to the acquisition of pro-inflammatory phenotype in psoriasis.

### Identification of ICOSLG–ICOS-mediated keratinocyte–T cell immunological synapse contact in psoriatic lesions

**Fig. 2:**
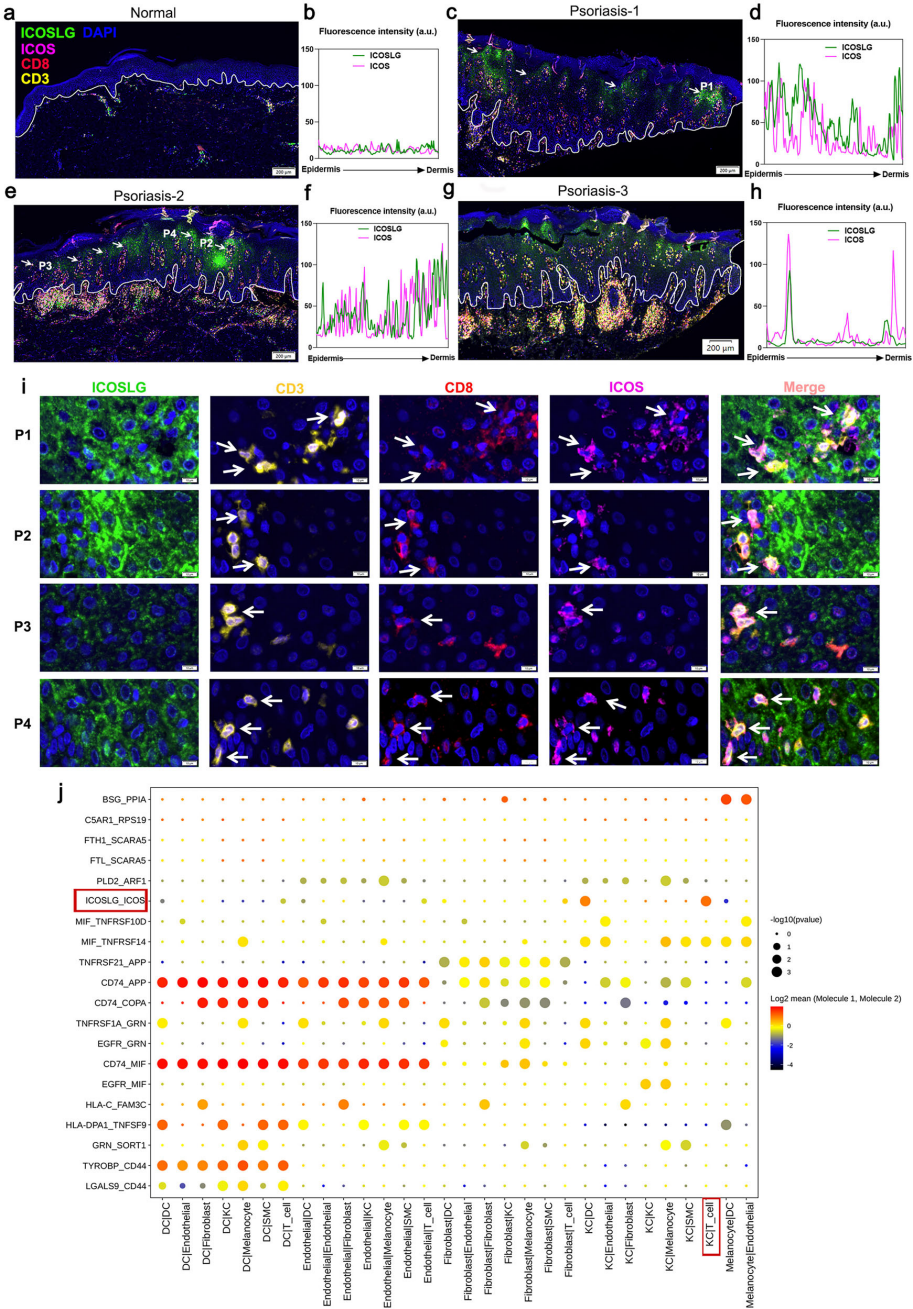
mIHC analysis and intercellular interaction between keratinocyte-derived ICOSLG and T cell surface-expressed ICOS of psoriatic skin lesions. **a**, **c**, **e**, **g** Representative multiplex immunohistochemistry (mIHC) staining images of normal skin and psoriatic lesions (*n* = 3 independent patients). Tissue sections were stained with anti-CD3, anti-CD8, anti-ICOS, and anti-ICOSLG antibodies. Scale bar, 200 μm. **b**, **d**, **f**, **h** Analysis of ICOSLG and RUNX1 immunofluorescence intensities corresponding to the staining images in panels **a**, **c**, **e** and **g** respectively. **i** mIHC staining revealed direct contact between keratinocyte-derived ICOSLG and T cell surface-expressed ICOS in psoriatic lesions. P1–P4 indicates the magnified views corresponding to the detailed areas in Fig. 1c, e. Scale bar, 10 μm. **j** Single-cell RNA-sequencing analysis identified interacting molecular pairs between distinct cell types. a.u. arbitrary unit, DAPI 4′,6-diamidino-2-phenylindole, DC dendritic cell, ICOSLG inducible T cell co-stimulator ligand, KC keratinocyte, SMC smooth muscle cell, t-SNE t-distributed stochastic neighbor embedding.

To validate the expression pattern of our scRNA-seq results in psoriatic lesional skin and further characterize keratinocyte–T cell interactions, we performed mIHC staining to analyze the local immune environment of human psoriasis skin versus healthy skin. Representative whole-tissue images revealed hyperplastic keratinocytes and infiltration of diverse immune cells in psoriatic skin. Notably, the epidermis of psoriasis skin exhibited robust infiltration of CD3^+^, CD8^+^, and ICOS^+^ cells (Fig. 2a, c, e, g). Spatial distribution analysis demonstrated a high colocalization efficiency between ICOSLG and ICOS immunofluorescence signals (Fig. 2b, d, f, h). In line with the RNA-seq and IHC evidence presented earlier, our immunofluorescence staining further confirmed the upregulation of ICOSLG in the psoriatic keratinocytes, with direct physical interaction observed between keratinocyte-derived ICOSLG and T cell surface-expressed ICOS (Fig. 2i).

To further analyze cellular intercommunication, we utilized CellPhoneDB^14^, a publicly available platform that integrates human or mouse scRNA-seq data with ligand–receptor signaling databases, to infer cell–cell communication. Our genomic analysis using this tool identified the ICOSLG–ICOS signaling axis as a key susceptibility pathway mediating interactions between keratinocytes and T cells in psoriasis (Fig. 2j). Together, these data demonstrated that keratinocytes engaged cutaneous-resident T cells via ICOSLG–ICOS signaling in psoriatic lesions.

### Keratinocyte-expressed ICOSLG functionally enhanced T cell activation

**Fig. 3:**
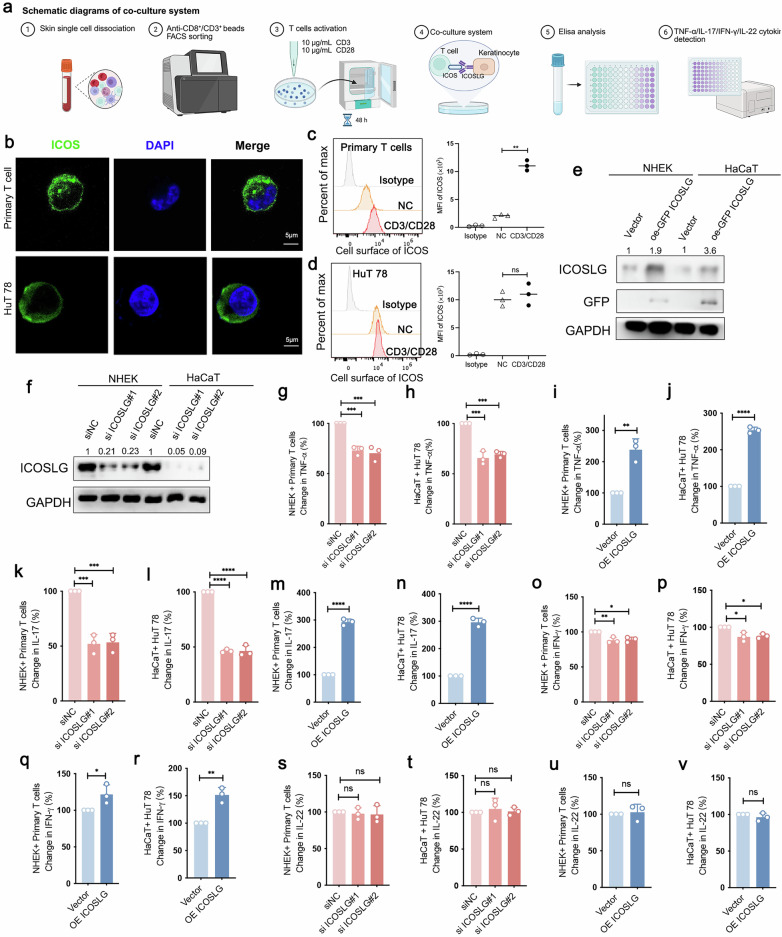
ICOSLG on keratinocyte mediates activation of T cells to secrete psoriasis-promoting cytokines. **a** Schematic diagram of the fluorescence-activated cell sorting (FACS) and co-culture experimental design. **b** Immunofluorescence staining of ICOS expression in activated primary T cells and HuT 78 cells (stimulated with 10 μg/ml anti-CD3 and anti-CD28 antibodies for 48 h). Scale bar, 5 μm. **c**, **d** Analysis of ICOS expression at cell surface of primary T cells and HuT 78 cells by flow cytometry following stimulation procedure. **e**, **f** Establishment of NHEK and HaCaT cell lines with ICOSLG knockdown or overexpression. **g**–**v** Co-culture of activated primary T cells and HuT 78 cells with ICOSLG knockdown or ICOSLG overexpression NHEK and HaCaT cells followed by ELISA analysis of cytokines (TNF-α, IFN-γ, IL-17A, and IL-22). Data were shown as mean ± SD of three independent experiments and were analyzed by two-tailed Student’s *t* test (parts **i**, **j**, **m**, **n**, **q**, **r**, **u**, and **v**) or one-way analysis of variance with Dunnett’s test (parts **c**, **d**, **g**, **h**, **k**, **l**, **o**, **p**, **s**, and **t**). *P < 0.05, **P < 0.01, ***P < 0.001, and ****P < 0.0001. Graphs were drawn by GraphPad Prism 10. Primary T cells (from three independent donors) were used in this study. DAPI 4′,6-diamidino-2-phenylindole, GFP green fluorescent protein, ICOSLG inducible T cell co-stimulator ligand, IFN-γ interferon-gamma, NC negative control, NHEK normal human epidermal keratinocyte, ns not significant, MFI mean fluorescence intensity, TNF-α tumor necrosis factor-alpha.

To investigate the functional role of ICOSLG, we constructed a co-culture system involving NHEKs and HaCaT cells co-cultured, respectively, with primary T cells and HuT 78 cells, followed by assessment of cytokine secretion post-co-culture (Fig. 3a). Immunofluorescence and flow cytometry assays were used to exam the similar surface antigen expression in primary T cells and HuT 78 cells. As shown, both primary T cells and HuT 78 cells expressed robust protein expression of ICOS (Fig. 3b–d), indicating their potential as surrogate cellular tools for functional validation. Epidermal keratinocytes, as critical components of skin-resident immunity, are known to induce and modulate T cell subsets that release TNF-α, IL-17, IFN-γ, and IL22, thereby amplifying psoriatic inflammation^7,15^. To validate the biological function of ICOSLG as a co-stimulatory signal in T cell activation and cytokine secretion, we knocked down or overexpressed ICOSLG in NHEK and HaCaT cells (Fig. 3e, f) and then co-cultured these cells with T cells. ELISA assay showed that silencing ICOSLG in NHEK and HaCaT cells significantly suppressed the secretion of TNF-α, IL-17, and IFN-γ, whereas overexpression of ICOSLG elevated the secretion of TNF-α, IL-17, and IFN-γ levels (Fig. 3g–r). Notably, no significant change was detected in IL-22 level (Fig. 3s–v). Altogether, our results demonstrated that keratinocytes regulated T cell activity through ICOSLG–ICOS signaling pathway.

### RUNX1 bound to ICOSLG promoter and enhanced ICOSLG expression in keratinocytes

**Fig. 4:**
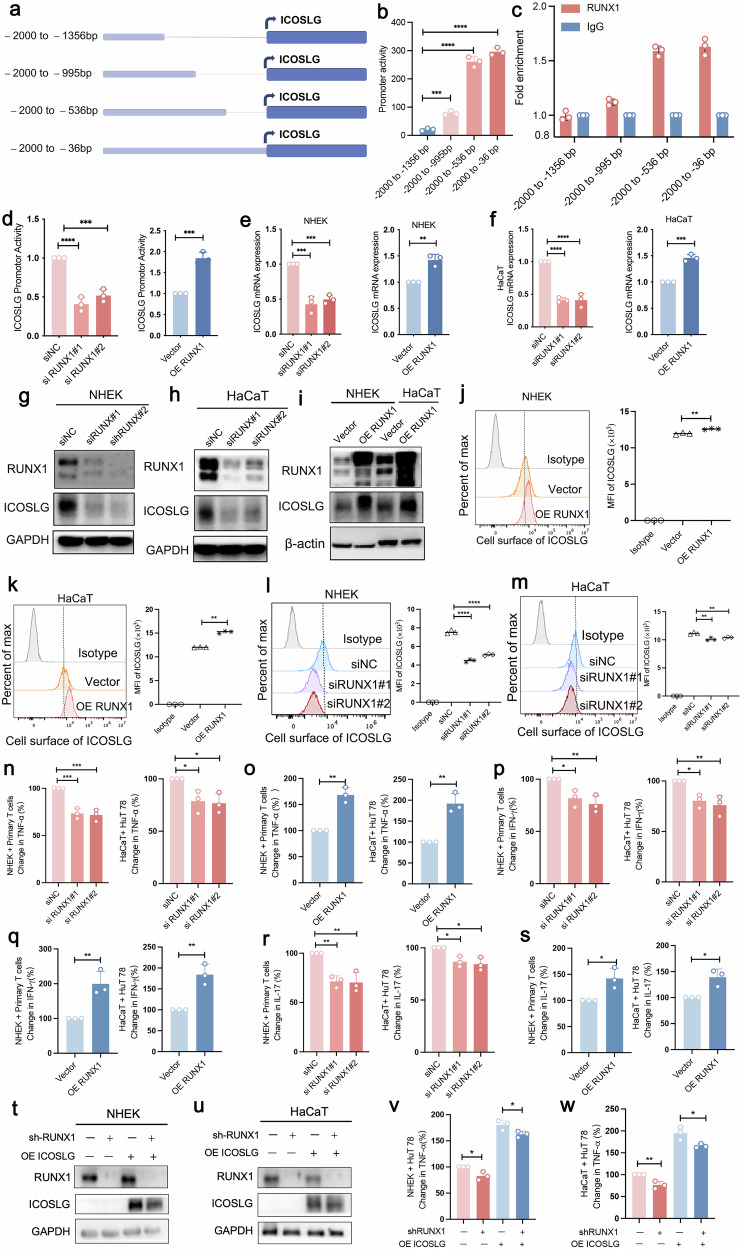
RUNX1 binds to the ICOSLG promoter to enhance ICOSLG expression. **a** The designated ICOSLG promoter fragments were cloned into pGL4.10 [luc2] reporter vectors (Promega) to generate luciferase reporter constructs. **b** Activity of the designated fragments of ICOSLG promoter was determined by dual-luciferase assay. Data from three independent experiments. **c** Binding of RUNX1 to ICOSLG promoter was measured by ChIP-qPCR. Data from three independent experiments. **d** Promoter activity of ICOSLG in NHEK cells transiently transfected with control siRNA (siNC), RUNX1 siRNAs (si-RUNX1-1 and si-RUNX1-2), control plasmid for RUNX1 (vector), or RUNX1 overexpression plasmid (OE RUNX1) was assessed by dual-luciferase assay. ICOSLG mRNA levels (parts **e** and **f**) and RUNX1 and ICOSLG protein expression (parts **g**–**i**). **j**–**m**, Cell surface ICOSLG in NHEK and HaCaT cells with transient RUNX1 knockdown or overexpression was determined. **n**–**s** Primary T cells and Hut 78 cells were co-cultured with NHEK and HaCaT cells in a 1:25 ratio for 48 h, and then TNF-α, IL-17A, IFN-γ, and IL-22 levels were detected by ELISA. **t**, **u** Western blot analysis of RUNX1 and ICOSLG expression in NHEK and HaCaT cells with RUNX1 knockdown and/or ICOSLG overexpression. **v**, **w** ELISA analysis of co-culture supernatant from RUNX1 knockdown and/or ICOSLG overexpression NHEK and HaCaT cells co-cultured with HuT 78 cells. Data were analyzed using two-tailed Student’s *t* test (parts **d**–**f**; blue panel; parts **j**, **k**, **o**, **q**, **s**, **v**, and **w**) or one-way analysis of variance (parts **d**–**f**; red panel; parts **b**, **l**–**n**, **p** and **r**) and presented as mean ± SD of three independent experiments; the indicated *P*-values are shown. Experiments were performed using primary T cells isolated from three independent healthy donors. ICOSLG inducible T cell co-stimulator ligand IFN-γ interferon-gamma NHEK normal human epidermal keratinocyte TNF-α tumor necrosis factor-alpha.

As our data showed earlier, ICOSLG and RUNX1 were upregulated in psoriasis keratinocytes, with a positive correlation between their expression levels. We hypothesize that RUNX1 is the distinct transcription factor for ICOSLG. To validate our hypothesis, we constructed luciferase reporter plasmids bearing various lengths of the core promoter region of ICOSLG (Fig. 4a). Dual-luciferase reporter assay showed that the most critical promoter region of ICOSLG was located in the −995 bp to −536 bp region (Fig. 4b). To confirm the results, we performed ChIP assay to validate our finding, and RUNX1 was shown to mainly bind at the −536 bp to −995 bp region of ICOSLG promoter (Fig. 4c). Consistently, knockdown of RUNX1 expression by siRNA decreased ICOSLG promoter activity, whereas overexpression of RUNX1 enhanced such process (Fig. 4d). Additionally, ICOSLG mRNA expression was also downregulated by RUNX1 knockdown in both NEHK and HaCaT cells, whereas it was upregulated by RUNX1 overexpression (Fig. 4e, f). To validate our findings at the protein level, we assessed ICOSLG expression in NEHK and HaCaT cells following RUNX1 perturbation. Western blot analysis further confirmed that RUNX1 silencing reduced ICOSLG protein expression in both NEHK and HaCaT cells (Fig. 4g, h). Conversely, RUNX1 overexpression increased ICOSLG protein level (Fig. 4i), corroborating its role in transcriptional regulation of ICOSLG.

Flow cytometry analysis demonstrated that RUNX1 knockdown reduced ICOSLG expression on the surface in both NHEK and HaCaT cells, whereas RUNX1 overexpression enhanced ICOSLG cell surface presentation (Fig. 4j–m and Supplementary Fig. 3a–d). To characterize the immunomodulatory properties of RUNX1, NHEK and HaCaT cells were co-cultured with primary T cells. ELISA quantification revealed that RUNX1 knockdown in keratinocytes significantly decreased T cell secretion of pro-inflammatory cytokines (TNF-α, IFN-γ, and IL-17) (Fig. 4n, p, r). Conversely, RUNX1 overexpression in keratinocytes increased the percentage of T cells that produced pro-inflammatory cytokines (Fig. 4o, q, s). Notably, RUNX1 knockdown in keratinocytes with ectopic ICOSLG expression (Fig. 4t, u) resulted in a partial reduction of CD25 and ki-67 expression and a concomitant downregulation of TNF-α, IFN-γ, and IL-17 secretion in co-cultured T cells (Fig. 4v, w and Supplementary Fig. 4a–f). Taken together, the findings indicate that high level of RUNX1 in keratinocytes promotes T cell activity through increasing ICOSLG expression.

### RUNX1 regulated ICOSLG transcription via recruiting GCN5 to the promoter in keratinocytes

**Fig. 5:**
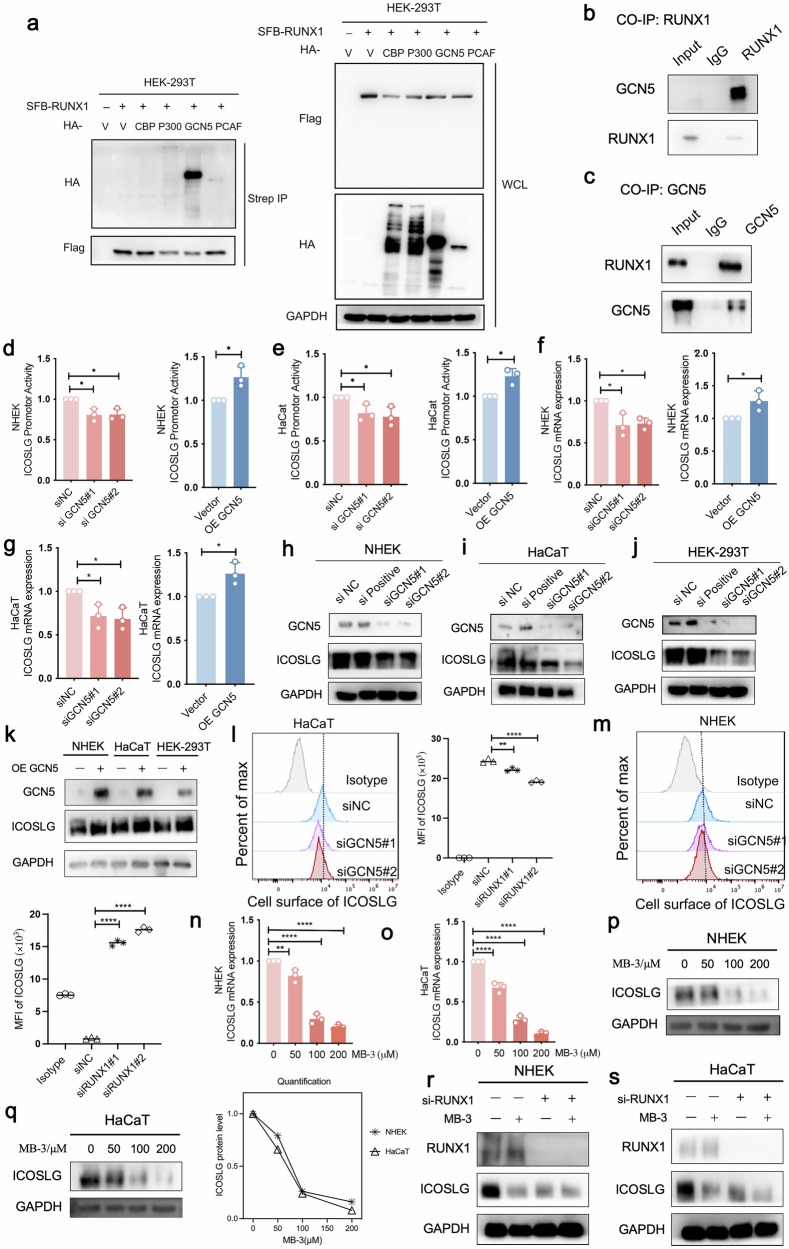
RUNX1 transcriptionally repressed ICOSLG expression through GCN5 recruitment. **a** Exogenous co-immunoprecipitation (co-IP) assay was conducted using HEK-293T cells, which were transfected with SFB-tagged RUNX1 and HA-tagged CBP, P300, GCN5, and PACF. Whole-cell lysates were immunoprecipitated with anti-Flag antibody (top) or anti-HA antibody (bottom), and the precipitates were determined using anti-HA antibody. Western blot analyses of whole-cell lysate and IP at the endogenous levels from NHEK cells using anti-RUNX1 (part **b**) or anti-GCN5 (part **c**) antibody. **d**, **e** Promoter activity of ICOSLG in NHEK and HaCaT cells transiently transfected with the indicated plasmids, measured by dual-luciferase assay. **f**, **g** ICOSLG mRNA levels in NHEK and HaCaT cells with transient GCN5 knockdown (red) or overexpression (blue) were examined by RT-qPCR. **h**–**k** Western blotting analysis of GCN5 and ICOSLG protein expression in NHEK, HaCaT, and HEK-293T cells with transient GCN5 knockdown or overexpression. **l**, **m** Analysis of ICOSLG expression at cell surface by flow cytometry following GCN5 knockdown. **n**, **o** ICOSLG mRNA expression in NHEK and HaCaT cells with MB-3 treatment for 12 h was detected by RT-qPCR. **p**, **q** Western blotting analysis of NHEK and HaCaT cells under MB-3 treatment at indicated drug concentrations. **r**, **s** NHEK and HaCaT cells transfected with control or RUNX1 siRNAs were treated with 100 μM MB-3 or vehicle (DMSO), and RUNX1 and ICOSLG protein levels were detected by western blotting. Data were presented as mean ± SD of three independent experiments and analyzed using two-tailed Student’s *t* test (parts **d**–**g**; blue panel) or one-way analysis of variance with Dunnett’s test (parts **d**–**g**; red panel; parts **l**–**o**). GCN5 general control non-derepressible 5, ICOSLG inducible T cell co-stimulator ligand, NHEK normal human epidermal keratinocyte, WCL whole-cell lysate.

Given the above results demonstrating that RUNX1 occupied the ICOSLG promoter and upregulated its transcription, we next sought to elucidate the mechanism by which RUNX1 facilitated ICOSLG at the transcriptional level. Histone acetyltransferase (HAT) functions primarily as a transcriptional activator and determines the level and dynamic balance of histone H3 acetylation, thereby regulating gene transcription. To identify HAT acetyltransferase potentially recruited by RUNX1 to the ICOSLG promoter, we co-transfected HEK-293T cells with SFB-tagged RUNX1 and HA-tagged HATs (CBP, P300, GCN5, and PCAF). Notably, GCN5 exhibited preferentially high-affinity binding to RUNX1 among these HATs (Fig. 5a). Consistent with these findings, endogenous co-IP assays verified the physical interaction between RUNX1 and GCN5 in keratinocytes (Fig. 5b, c). To validate the effect of GCN5 on ICOSLG, we used distinct siRNAs to disrupt GCN5 expression in keratinocytes. Knockdown of GCN5 reduced ICOSLG promoter activity, whereas GCN5 overexpression enhanced it (Fig. 5d, e). RT-qPCR assay showed that knockdown of GCN5 decreased the mRNA level of ICOSLG in both NHEK and HaCaT cells, whereas overexpression of GCN5 upregulated the mRNA level of ICOSLG (Fig. 5f, g). Likewise, western blotting analysis showed that depletion of GCN5 caused substantial suppression of ICOSLG protein in NHEK (Fig. 5h), as well as in HaCaT and 293T cells (Fig. 5i, j). Conversely, ectopic expression of GCN5 increased ICOSLG protein level (Fig. 5k). Consistently, flow cytometry analysis of surface ICOSLG expression further confirmed that GCN5 silencing reduced ICOSLG surface expression (Fig. 5l, m and Supplementary Fig. 3e,f).

To further validate the functional role of GCN5 in ICOSLG regulation, we used butyrolactone 3 (MB-3), a selective small-molecule inhibitor targeting the HAT GCN5. RT-qPCR analysis of NHEK and HaCaT cells treated with MB-3 showed that MB-3 downregulated ICOSLG mRNA level (Fig. 5n, o). As Fig. 5p, q showed, MB-3 suppressed the protein level of ICOSLG in both NHEK and HaCaT cells in a dosage-dependent manner. In the meanwhile, more significant inhibition of ICOSLG protein level on control group was observed under the MB-3 treatment compared with those on its RUNX1 knockdown (Fig. 5r, s). Cell proliferation assays demonstrated that MB-3, across a range of concentrations from 50 µM to 200 µM, did not significantly affect the growth of NHEK or HaCaT cells (Supplementary Fig. 5a, b). Collectively, these results demonstrated that RUNX1 recruited GCN5 to ICOSLG promoter, thereby determining ICOSLG at the transcriptional level.

### GCN5 inhibitor MB-3 attenuated psoriasiform dermatitis in vivo

**Fig. 6:**
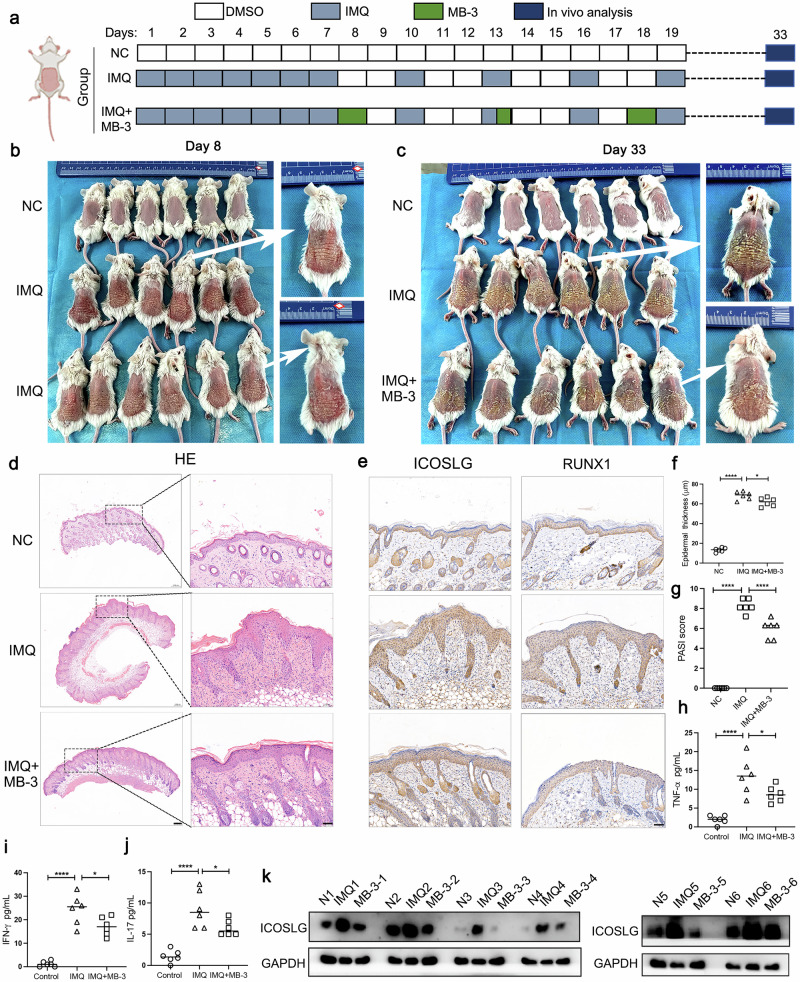
GCN5 inhibitor MB-3 attenuated psoriasiform dermatitis in vivo. **a**–**c** A psoriasiform mouse model was established by daily topical application of 5% imiquimod cream (IMQ, 62.5 mg per mouse) on the shaved dorsal skin of 6–8-week-old female BALB/c mice for 7 consecutive days. The inflammatory response peaked at days 6 and 7. To maintain psoriasiform inflammation, IMQ was administered topically every 3 days. Histone deacetylase inhibitor (MB-3) was injected intraperitoneally every 5 days at a dose of 5 mg/kg. Mice were euthanized, and skin lesions were collected at day 33. **d**, **e** HE and immunohistochemical (IHC) staining of the indicated mouse skin tissues. Scale bar, 0.2 mm (HE) and 0.05 mm (IHC). **f**, **g** Epidermal thickness and PASI scores of the indicated mouse groups. **h**–**j** ELISA analysis of TNF-α, IL-17A, and IFN-γ levels in mouse skin tissues. **k** Western blotting analysis of ICOSLG in indicated mouse skin tissues. Data were presented as mean ± SD of three independent experiments and analyzed by one-way analysis of variance with Tukey's correction. HE hematoxylin and eosin ICOSLG inducible T cell co-stimulator ligand IFN-γ interferon-gamma PASI Psoriasis Area and Severity Index TNF-α tumor necrosis factor-alpha.

To further investigate the in vivo functional contribution of ICOSLG to psoriasis, we established a mouse model of psoriasiform dermatitis. Imiquimod was topically applied to the dorsal skin of mice for 7 consecutive days (62.5 mg per day), with psoriasiform dermatitis developing at the 8th day. Subsequently, mice were intraperitoneally injected with 5 mg/kg butyrolactone 3 (MB-3) every 5 days, and skin tissues were collected following euthanasia at approximately day 33 (Fig. 6a–c). Assessment of serum ALT/AST levels and liver histology showed no significant difference in hepatotoxicity between any experimental group and controls (Supplementary Fig. 5c–e). No statistically significant differences were observed in body weight growth curves among all groups of mice (Supplementary Fig. 5f–h). The animal study demonstrated that MB-3 alleviated the progression of psoriasiform lesions (Fig. 6b, c). We further examined the expression of ICOSLG and pro-inflammatory cytokines in psoriasiform lesions using IHC and ELISA analysis. Experimental data demonstrated that ICOSLG expression was upregulated in psoriasiform lesions compared with control groups, and MB-3 treatment partially reversed this upregulation (Fig. 6d, e), thereby leading to a reduction in epidermal thickness and PASI score within the psoriatic lesions (Fig. 6f, g). Notably, MB-3 treatment suppressed the level of local cytokine (TNF-α, IFN-γ, and IL-17) in psoriasiform lesions (Fig. 6h–j). Western blotting assays further confirmed that MB-3 also reduced the expression of ICOSLG in protein level (Fig. 6k). Together, our in vivo study supported that MB-3 attenuated the psoriasiform development by depressing ICOSLG expression and psoriatic lesion inflammation.

### GCN5 inhibitor MB-3 decelerated the recurrence of psoriasiform lesions

**Fig. 7:**
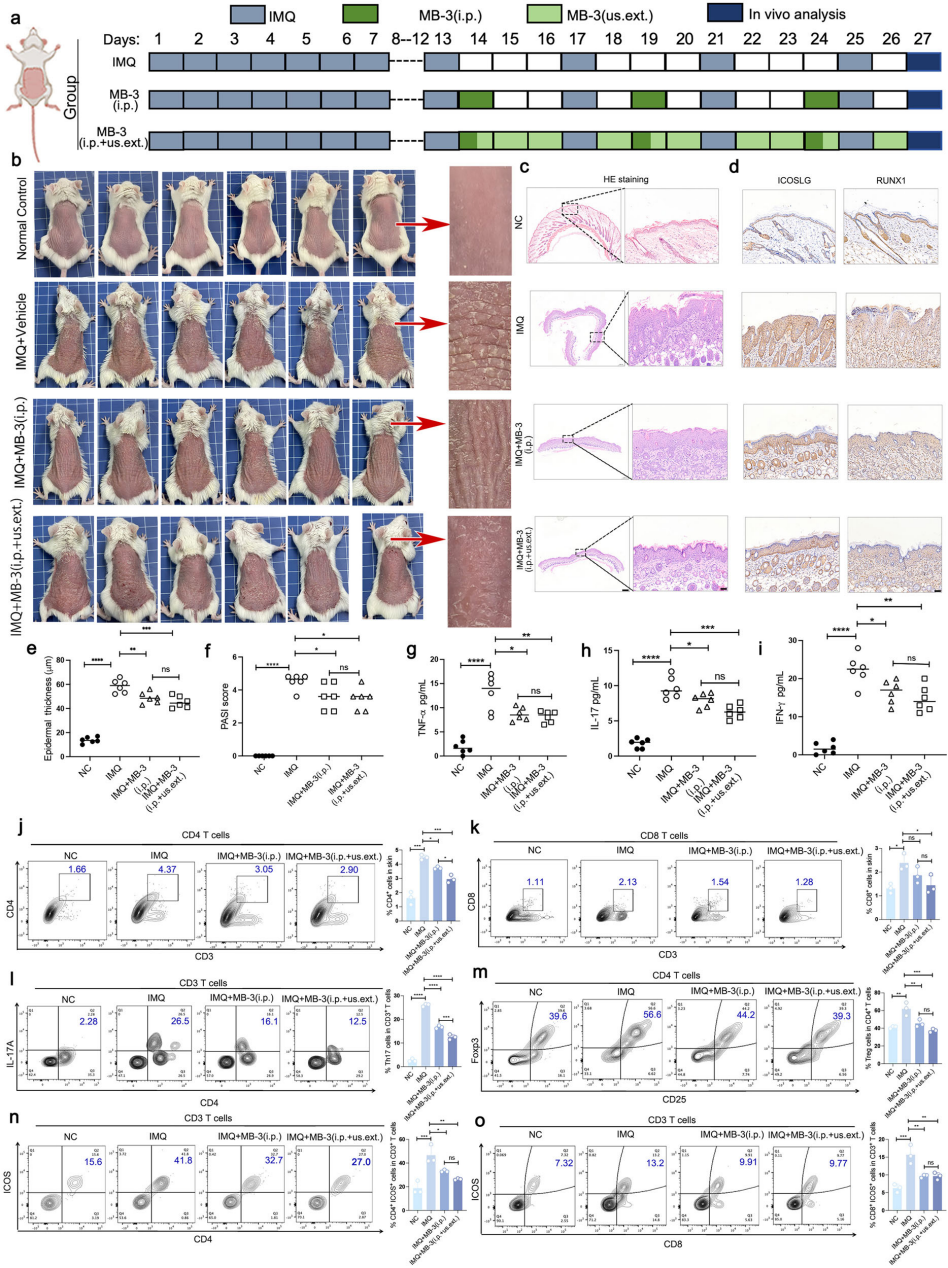
MB-3 suppressed disease recurrence in imiquimod-induced psoriasiform model. **a** Animal experimental flowchart. **b** Representative lesion images from: NC, IMQ, IMQ + MB-3 (i.p.), and IMQ + MB-3 (i.p.+us.ext.). **c**, **d** Representative HE and immunohistochemical (IHC) staining sections of dorsal skin from the indicated groups. Scale bar, 0.2 mm (HE) and 0.05 mm (IHC). **e**, **f** Epidermal thickness and PASI score measurements of the indicated mouse group. **g**–**i** ELISA quantification of TNF-α, IL-17A, and IFN-γ levels in cutaneous lysates across experimental cohorts. Flow cytometric analysis of skin single-cell suspensions with: anti-CD3/CD4 (part **j**); anti-CD3/CD8 (part **k**); anti-CD3/CD4/IL-17A (part **l**); anti-CD4/CD25/Foxp3 (part **m**); anti-CD3/CD4/ICOS (part **n**); anti-CD3/CD8/ICOS (part **o**). Data are presented as mean ± SD of three independent experiments and analyzed by one-way analysis of variance with Tukey's correction. *P < 0.05, **P < 0.01, ***P < 0.001, and ****P < 0.0001, and ns represents no statistical significance. Graphs were drawn by GraphPad Prism 10. HE hematoxylin and eosin ICOSLG inducible T cell co-stimulator ligand IFN-γ interferon-gamma IMQ imiquimod PASI Psoriasis Area and Severity Index TNF-α tumor necrosis factor-alpha.

To determine the in vivo functional contribution of MB-3 to psoriasiform recurrence, we designed a temporal intervention study to evaluate both the timing and severity of psoriasiform lesion recurrence using our established psoriasiform mouse model (Fig. 7a). Psoriasiform lesions were first induced by daily application of IMQ for 7 consecutive days. Following a 5-day treatment cessation period, IMQ was re-administered on day 13 to trigger recurrence. In the follow-up experiment, IMQ was applied triweekly (q3d) to induce psoriasiform lesions. To evaluate the impact of drug delivery routes, MB-3 was administered either through intraperitoneal injection (i.p., q3d) or via a dual-route approach combining i.p. and topical delivery (i.p.+ us.ext.). Our animal studies revealed that MB-3 treatment not only reduced the general inflammation of psoriasiform lesions but also postponed their emergence (Fig. 7b and Supplementary Fig. 6a,c). Parallel IHC evaluation revealed that MB-3-mediated psoriasiform alleviation correlated with inhibition of cutaneous ICOSLG expression (Fig. 7c, d) and resulted in decreased epidermal thickness and PASI scores with MB-3 treatment (Fig. 7e, f). Notably, combined i.p. and topical MB-3 administration demonstrated superior efficacy in lesion resolution compared with intraperitoneal monotherapy. Local skin cytokine profiling by ELISA confirmed that MB-3 administration potently inhibited pathogenic psoriatic factors (TNF-α, IFN-γ, and IL-17) at lesion sites (Fig. 7g–i). To further characterize the immunological impact, single-cell suspensions prepared from harvested skin tissues were analyzed by multiparametric flow cytometry. Our flow cytometry data showed that MB-3 treatment reduced the population of CD3^+^CD4^+^-positive cells compared with the control group (Fig. 7j); also a slight reduction in the proportion of CD3^+^CD8^+^ cells between the MB-3-treated and IMQ groups was observed (Fig. 7k). Meanwhile, our single-cell flow cytometry analysis demonstrated that MB-3 treatment led to a marked reduction in pathogenic CD3^+^CD4^+^IL-17A^+^ (T_H_17) cells in psoriasis lesions (Fig. 7l) and, consequently, to a decline in the frequency of CD4^+^CD25^+^Foxp3^+^ T_reg_ cells (Fig. 7m). Consistently, our flow cytometry analysis revealed that the majority of both CD4^+^ and CD8^+^ T cells expressed high levels of ICOS, supporting that ICOSLG serves as a key ligand for their activation, and administration of MB-3 significantly reduced the proportion of ICOS^+^ cells within both CD4^+^ and CD8^+^ T cell compartments (Fig. 7n, o). These findings indicated that MB-3 alleviates recurrent psoriasis inflammation, which is potentially mediated through the inhibition of local immune responses in the skin.

### GCN5 is required for MB-3-mediated inhibition of the ICOS/ICOSLG co-stimulatory pathway in vivo

**Fig. 8:**
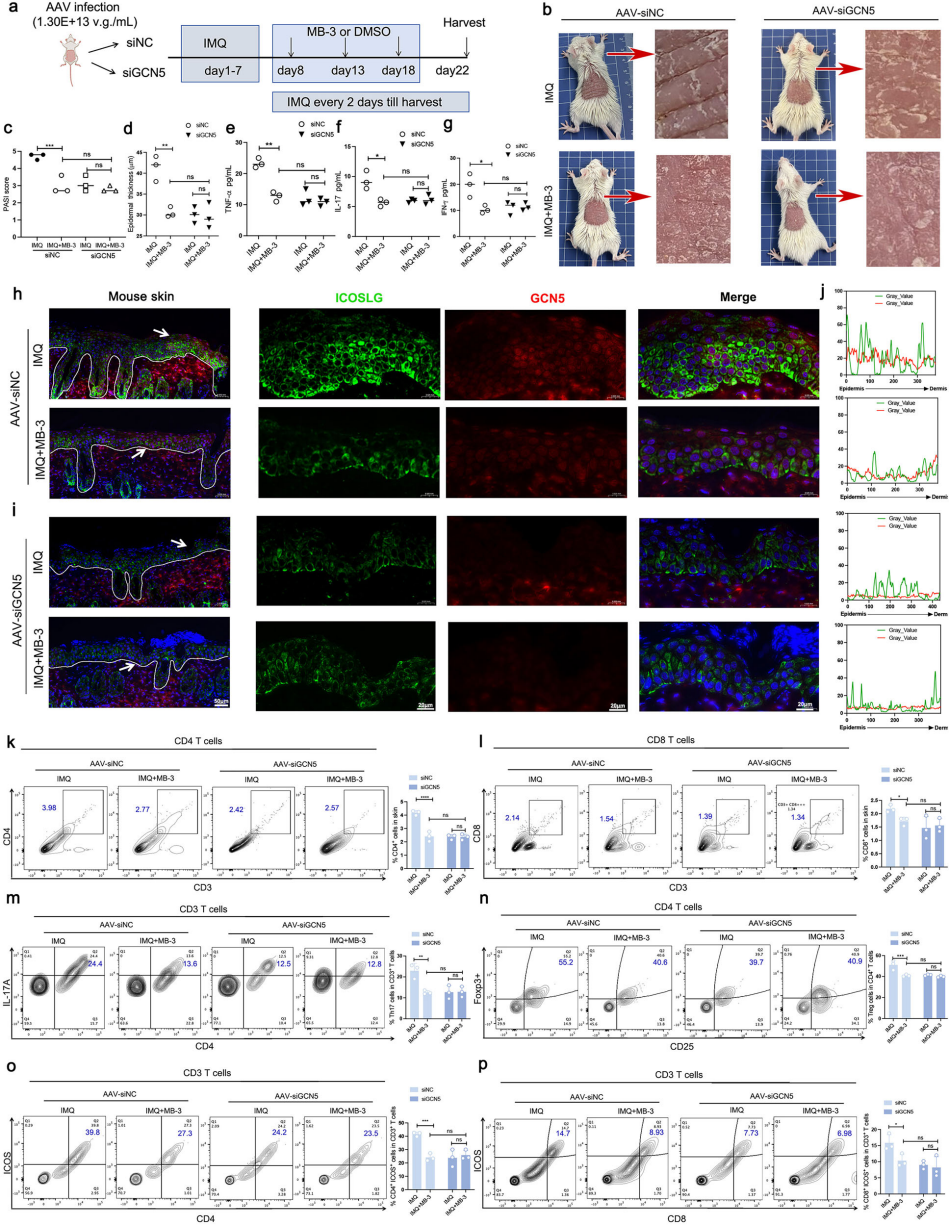
GCN5 is required for the function of MB-3 in its therapeutic effects in psoriasis. **a** Animal experimental flowchart. **b** Representative lesion images from: siNC + IMQ, siNC + IMQ + MB-3, siGCN5 + IMQ, and siGCN5 + IMQ + MB-3. **c**, **d** Quantification of epidermal thickness and PASI scores in the indicated groups. **e**–**g** Analysis of TNF-α, IL-17A, and IFN-γ levels in skin lysates was performed by ELISA across all experimental groups. **h**, **i** Representative mIHC staining sections of dorsal skin from the indicated groups. Tissue sections were stained with anti-GCN5 and anti-ICOSLG antibodies. Scale bar, 0.05 mm (left) and 0.02 mm (right). **j** Analysis of ICOSLG and GCN5 immunofluorescence intensities corresponding to the staining images in panels **h** and **i** respectively. Flow cytometric analysis of skin single-cell suspensions was performed to identify major T cell populations and subsets, using the following antibody panels: anti-CD3/CD4c (part **k**); anti-CD3/CD8 (part **l**); anti-CD3/CD4/IL-17A (part **m**); anti-CD4/CD25/Foxp3 (part **n**); anti-CD3/CD4/ICOS (part **o**); and anti-CD3/CD8/ICOS (part **p**). Data are mean ± SD (*n* = 3 mice per group). One-way analysis of variance with Tukey's correction. *P < 0.05, **P < 0.01, ***P < 0.001, ****P < 0.0001, ns (not significant). AAV adeno-associated virus; DMSO dimethyl sulfoxide; GCN5 general control non-derepressible 5 ICOSLG inducible T cell co-stimulator ligand IFN-γ interferon-gamma IMQ imiquimod PASI Psoriasis Area and Severity Index TNF-α tumor necrosis factor-alpha.

To determine whether the therapeutic effect of MB-3 is GCN5-dependent, we established a keratinocyte-specific AAV-mediated GCN5 knockdown mouse model. As expected, although this genetic intervention alleviated imiquimod-induced psoriasiform skin lesions, it concurrently impaired the therapeutic efficacy of MB-3 (Fig. 8a, b and Supplementary Fig. 6b, d). Specifically, psoriasiform lesions in the GCN5 knockdown group showed no significant improvement in response to MB-3 treatment compared with the control group. Consistent with this, no statistically significant differences were observed in PASI scores or epidermal thickness in GCN5 knockdown mice with or without MB-3 therapy (Fig. 8c, d). Upon MB-3 treatment, TNF-α, IFN-γ, and IL-17 levels in skin lesions of GCN5-knockdown mice remained comparable to those in the control group (Fig. 8e–g). mIHC revealed an efficient reduction of GCN5 protein in keratinocytes within psoriatic lesions following AAV-mediated knockdown, along with a corresponding decrease in ICOSLG protein levels (Fig. 8h–j). Next, single-cell suspensions were prepared for immunophenotyping to evaluate local immune infiltration following MB-3 therapy. Compared with the siNC group, AAV-mediated GCN5 knockdown in keratinocytes significantly decreased total CD4⁺ T cells and CD8^+^ T cells — including T_H_17, T_reg_ subsets as well as ICOS⁺ CD4⁺ and ICOS⁺ CD8⁺ T cells (Fig. 8k–p). Notably, following this knockdown, MB-3 failed to reduce the proportions of total CD4^+^ and CD8^+^ T cells (Fig. 8k,l), ICOS^+^ CD4^+^ and CD8^+^ T cells (Fig. 8o,p) as well as T_H_17 cells and T_reg_ cells (Fig. 8m,n) within the psoriatic lesions. Taken together, our findings demonstrate that GCN5 is required for MB-3-mediated inhibition of the ICOS/ICOSLG co-stimulatory pathway in vivo.

## Discussion

Elevated expression of the co-stimulatory receptor ICOS is a hallmark of tissue-resident T cells in both mouse and human studies^10,16^. ICOS gene deletion or blockade of its ligand ICOSLG severely impairs the establishment of these cells. Mechanistic investigations have revealed that the ICOS–PI3K signaling pathway has a crucial role in the efficiency of T cell tissue residency^12^. Further studies confirm that both WT and *ICOS*^–^/^–^ P14 T cells fail to efficiently establish tissue-resident T cells in an ICOSLG-deficient environment, demonstrating that ICOS–ICOSLG signal is required for the generation of tissue-resident T cells^12^. ICOSLG is abundantly expressed on various APCs, and single-cell mouse cell atlas analyses indicate robust ICOSLG transcription in certain non-hematopoietic cells within non-lymphoid tissues^13^. Activation of the ICOS–ICOSLG signaling pathway has a pivotal role in supporting the efficient generation and function of CD8^+^ tissue-resident T cells^12^. Cutaneous-resident T cells are recognized as one of the primary drivers of inflammatory memory and disease recurrence in psoriasis^3,17^. However, the precise cellular subsets expressing co-stimulatory molecule of ICOSLG and orchestrating the maintenance of tissue-resident T cell populations in cutaneous microenvironments remain uncharacterized.

Here, we used scRNA-seq to characterize the cellular landscape and transcriptional profiles within the immune microenvironment of psoriatic lesions. By analyzing differential gene expression and pathway enrichment, we found that upregulated genes (more than twofold; *P* < 0.01) in keratinocytes from the psoriatic group exhibited strong immune responses, particularly in T cell-mediated immunity. Further investigation into the expression profiles and composition of keratinocytes revealed that ICOSLG was significantly overexpressed in keratinocytes from psoriatic lesional tissues. Our study thus identified a candidate cell subtype potentially dominating ICOSLG expression.

The binding of ICOSLG to ICOS mediates co-stimulatory signals in T cells, thereby enhancing T cell activation and proliferation^18^. ICOSLG has a crucial role in mediating local tissue responses to inflammatory conditions by co-stimulating T cell functions and regulating secondary immune responses^19,20^. Thus, we seek to elucidate the precise interaction mechanisms between keratinocyte-expressed ICOSLG and T cells. Through analysis of scRNA-seq cell–cell interactions, we discovered that in patients with psoriasis, keratinocytes interact with T cells via ICOSLG binding to ICOS on the T cell surface (as shown in Fig. 2). Therefore, the precise molecular mechanisms through which this binding event initiates localized T cell immune activation will form a critical line of inquiry in our ongoing research.

Increased T cell infiltration into psoriatic lesions has been observed in patients with psoriasis^3^. Using mIHC assay, we revealed the presence of an impressive population of CD3^+^CD8^+^ICOS^+^ T cells in the epidermis of psoriasis lesions (Fig. 2). Notably, the immunofluorescence staining demonstrated that keratinocytes highly expressed ICOSLG and spatially colocalized with CD3^+^CD8^+^ICOS^+^ T cells in the psoriatic immune microenvironment, further supporting the existence of direct interactions between keratinocytes and cutaneous-resident T cells. This observation raises the question: what specific immune responses are triggered by direct contact between CD3^+^CD8^+^ICOS^+^ T cells and keratinocytes within this local microenvironment? Upon co-culture of primary T cells or HuT 78 with keratinocytes, we observed that ICOSLG knockdown significantly suppressed the production of inflammatory cytokines (including IL-17, TNF-α, and IFN-γ). Conversely, ICOSLG overexpression enhanced the secretion of these pro-inflammatory mediators. These findings indicate that keratinocytes may activate locally resident T cells in the epidermal microenvironment through the ICOSLG–ICOS axis, thereby promoting the secretion of psoriasis-associated cytokines.

A growing body of evidence suggests that inflammatory memory exists not only in adaptive immune cells such as T and B cells but also in innate immune cells and certain non-immune cells^21,22^. Recently, Fuchs and co-workers demonstrated that stem-like basal keratinocytes are also capable of acquiring long-term memory of inflammation. Keratinocytes serve as the central effectors driving the psoriatic inflammatory cascade^21^. On the basis of single-cell transcriptomic profiling and IHC validation, our clinical findings identified ICOSLG and RUNX1 as significantly upregulated genes in psoriasis keratinocytes, with a positive correlation between their expression levels. Integrating the evidence in previous study and our findings, we hypothesize that the distinct expression profile of keratinocytes in psoriasis may underlie their capacity to induce localized immune responses and establish immune memory. More well-designed studies are needed to understand the mechanism inside.

Functioning as a master transcriptional regulator, RUNX1 orchestrates diverse physiological and pathological processes from embryogenesis to tumor development via epigenetic control of histone acetylation^23–25^. Nevertheless, the mechanism by which elevated RUNX1 links ICOSLG mRNA expression in psoriasis keratinocytes remains to be determined. Notably, we found that RUNX1 promoted the expression of ICOSLG at both transcriptional and protein levels. In addition, the dual-luciferase reporter assay has identified the core ICOSLG promoter region bound by RUNX1 as spanning −995 bp and −536 bp, which was further validated by flag bead co-IP and Chip-qPCR experiments. Hence, our study elucidates the transcriptional mechanisms underlying elevated surface antigen expression of ICOSLG in keratinocytes from psoriatic lesions.

GCN5 is a highly conserved lysine acetyltransferase (KAT) belonging to the GNAT (GCN5-related *N*-acetyltransferase) superfamily. It has a pivotal role in epigenetic regulation by catalyzing the acetylation of histones, thereby modulating chromatin structure and gene transcription^26–28^. Recent study reported the involvement of GCN5 in inflammatory and immune responses. It modulates the acetylation of transcription factors such as NF-κB and STAT3, thereby influencing cytokine production and immune cell activation^29^. This positions GCN5 as a potential therapeutic target for autoimmune diseases and chronic inflammatory conditions, although further research is needed to unravel its complex mechanisms in skin tissue-specific pathologies. Here, we demonstrate that RUNX1 promotes the transcription of ICOSLG by recruiting GCN5 to its promoter, thereby enhancing T cell activation and cytokine production. Our research delineated the transcriptional machinery responsible for pathological ICOSLG overexpression in psoriatic keratinocytes at molecular resolution.

As a potent modulator of GCN5-mediated lysine acetylation, MB-3 serves as a valuable research tool for investigating the role of GCN5 in cancer, metabolic disorders, autoimmune diseases, and neurological conditions^30,31^. To date, there has been no evidence that MB-3 alleviates skin inflammation, decreases T cell infiltration, or induces immunosuppression by downregulating T cell co-stimulatory molecule expression. Our study shows that MB-3 curbs psoriasis pathogenesis and relapse, likely through suppressing local cutaneous immunity, as evidenced by reduced local lesional cytokine levels, infiltration of CD4^+^ T cells (including T_H_17 and T_reg_ cells), and a significant decrease in the number of ICOS^+^ T cells. Significantly, our keratinocyte-specific GCN5 knockdown mouse model showed that GCN5 is required for the function of MB-3 in its therapeutic effects in psoriasis. On the basis of the aforementioned data, we reasoned that alterations in cytokine levels within psoriatic lesions after MB-3 treatment are associated with the quantity of T cells. To distinguish whether the reduced cytokine levels are caused by decreased T cell number or impaired T cell activation, we provide a theoretical analysis based on the known biological functions of the GCN5–RUNX1–ICOSLG axis and the mechanism of MB-3 action. MB-3 acts as a specific inhibitor of GCN5, which blocks the formation of the RUNX1–GCN5 complex and subsequently downregulates ICOSLG expression in keratinocytes. Given that ICOSLG–ICOS interaction is critical for both the survival and infiltration of effector T cells into psoriatic lesions, as well as the maintenance of their activated status and cytokine-secreting capacity, the reduced cytokine concentrations observed after MB-3 treatment are likely attributable to both scenarios. Given the immunomodulatory role of the GCN5–RUNX1–ICOSLG axis in psoriasis, targeting this pathway with inhibitors could be developed as part of a combination strategy with monoclonal antibodies to enhance the efficacy and to prevent recurrence. This area calls for more comprehensive and well-designed researches.

In summary, through orthogonal validation with transcriptomics, psoriasis specimen, cell experiments, and animal model, we have identified the RUNX1–GCN5 transcriptional axis that drives ICOSLG overexpression via chromatin remodeling at the ICOSLG promoter in psoriatic keratinocytes. Notably, pharmacological inhibition of GCN5 using the selective inhibitor MB-3 attenuated the progression and recurrence of psoriasis through immunosuppression via ICOS–ICOSLG signal. This study elucidates how ICOSLG-mediated keratinocytes–T cell crosstalk in psoriatic lesions orchestrates local immune activation and drives disease progression. These findings establish a mechanistic framework for future investigations into psoriatic pathogenesis and uncover novel therapeutic targets for disease intervention and relapse prevention.

## Supplementary information

Supplementary figures

## Data Availability

The raw sequence data reported in the present paper have been deposited in the GEO database under accession no. GSE248121. The data used or analyzed in this study are available from the corresponding author on reasonable request.
