# Supplementary material for: RUNX1 recruitment of GCN5 in keratinocytes upregulates ICOSLG and promotes T cell activation in the psoriasis microenvironment
Source: Exp Mol Med. 2026 Jun 4;58(6):1838–53. doi: 10.1038/s12276-026-01738-8 (PMC13324650; doi:10.1038/s12276-026-01738-8)
Supplement: Supplementary file 1 — Supplementary figures [file 12276_2026_1738_MOESM1_ESM.pdf]

Supplementary Figures

Supplementary Fig. 1

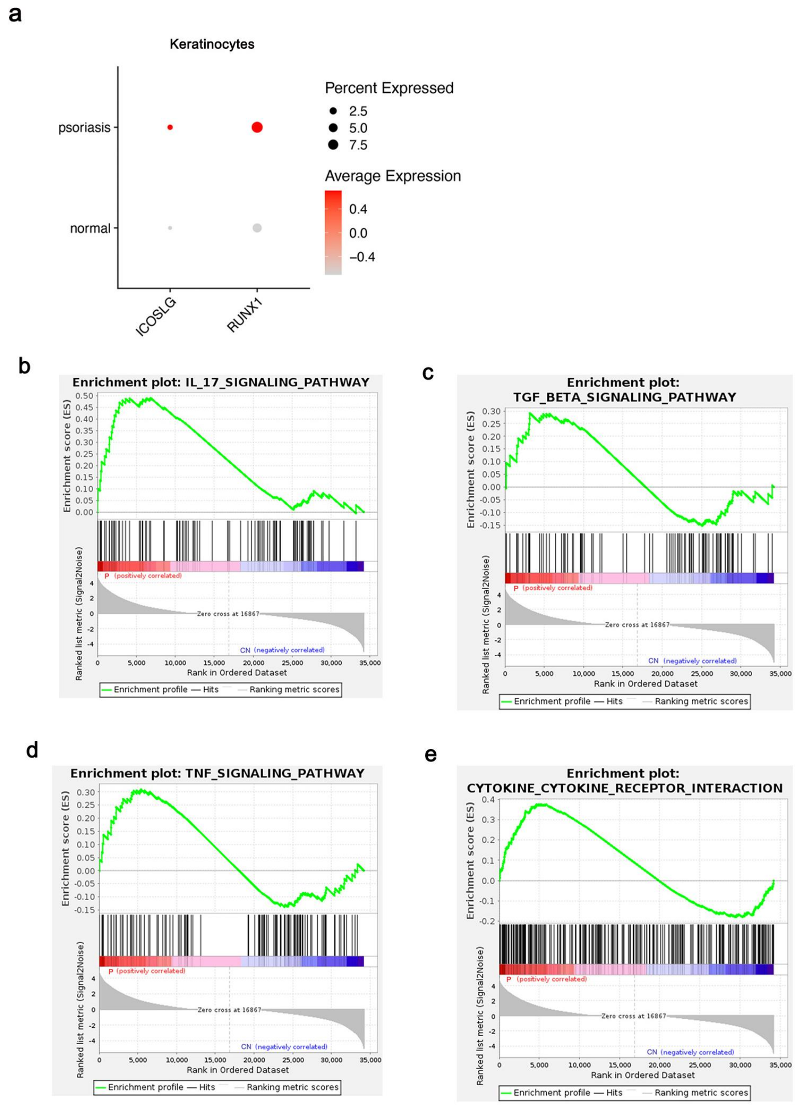

Supplementary Fig. 1 ICOSLG and RUNX1 expression in psoriatic lesions and visualization of GSEA enrichment results.

(a) ICOSLG and RUNX1 expression between psoriasis and normal skin keratinocytes. Data were downloaded from GSE230842 and GSE237767. (b-e) Gene set enrichment analysis (GSEA) of keratinocytes cell gene signatures using the transcriptome of psoriasis tissues versus control samples.

Supplementary Fig. 2

a

Clinical information of psoriasis donors

Patient	Gender	Age	Donor site	Diagnose
1	M	21	Arm	Plaque Psoriasis
2	M	25	Thigh	Plaque Psoriasis
3	M	20	Arm	Plaque Psoriasis
4	F	68	Chest	Plaque Psoriasis
5	F	50	Back	Plaque Psoriasis
6	M	82	Shank	Plaque Psoriasis
7	M	72	Shank	Plaque Psoriasis
8	F	45	Arm	Plaque Psoriasis
9	M	62	Thigh	Plaque Psoriasis
10	F	38	Back	Plaque Psoriasis
11	M	45	Arm	Plaque Psoriasis
12	F	43	Back	Plaque Psoriasis
13	M	52	Arm	Plaque Psoriasis
14	M	50	Chest	Plaque Psoriasis
15	M	67	Back	Plaque Psoriasis
16	F	56	Abdomen	Plaque Psoriasis
17	M	67	Shank	Plaque Psoriasis

b

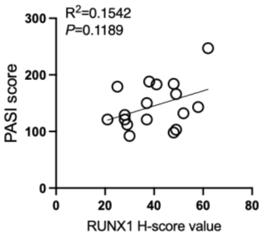

Supplementary Fig. 2 Clinical characteristics of the included psoriasis patients.

(a) Clinical information of psoriasis patients included in the study. (b) Spearman correlation analysis was performed to assess the relationship between the expression levels of RUNX1 in lesional skin and the corresponding PASI scores.

Supplementary Fig. 3

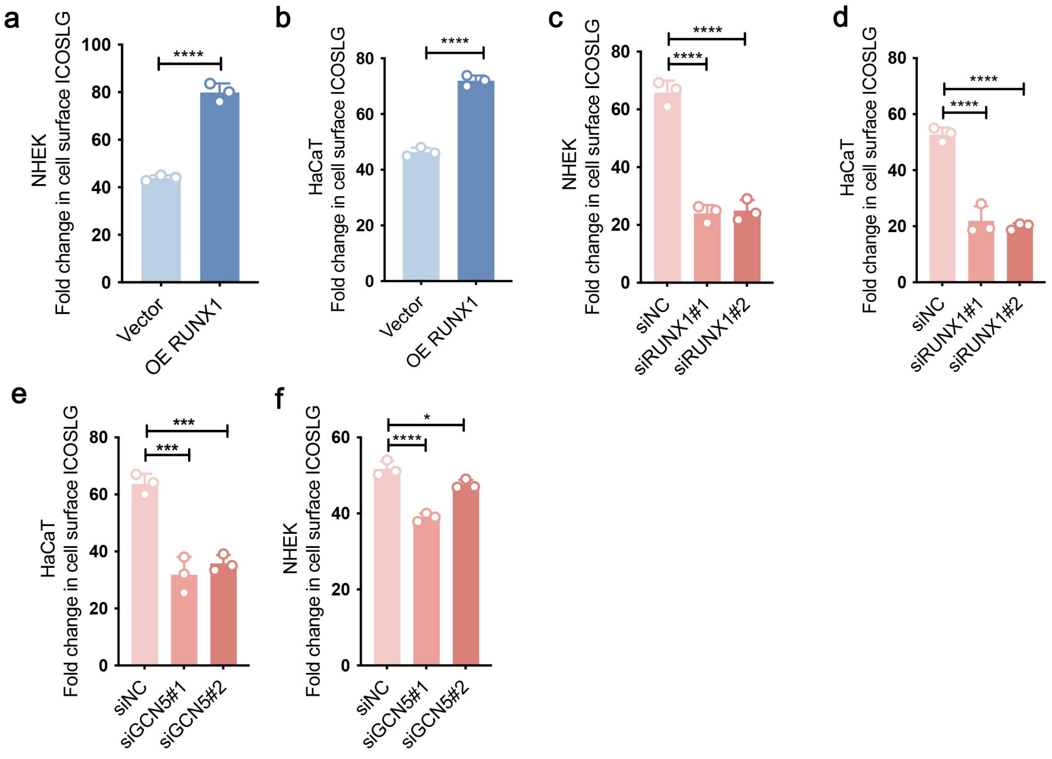

Supplementary Fig. 3 RUNX1 and GCN5 are involved in the regulation of T cell activation
(a-d) ICOSLG staining in NHEK and HaCaT cells with knockdown or overexpression of RUNX1, statistical analysis of ICOSLG$^+$ cells. (e-f) ICOSLG staining in NHEK and HaCaT cells with knockdown or overexpression of GCN5. Data are mean ± SD (n=3 independent experiments). Two-tailed Student's t-test (a-b) and one way ANOVA with Dunnett test (c-f); *$P < 0.05$, **$P < 0.01$, ***$P < 0.001$, ****$P < 0.0001$, ns (not significant).

Supplementary Fig. 4

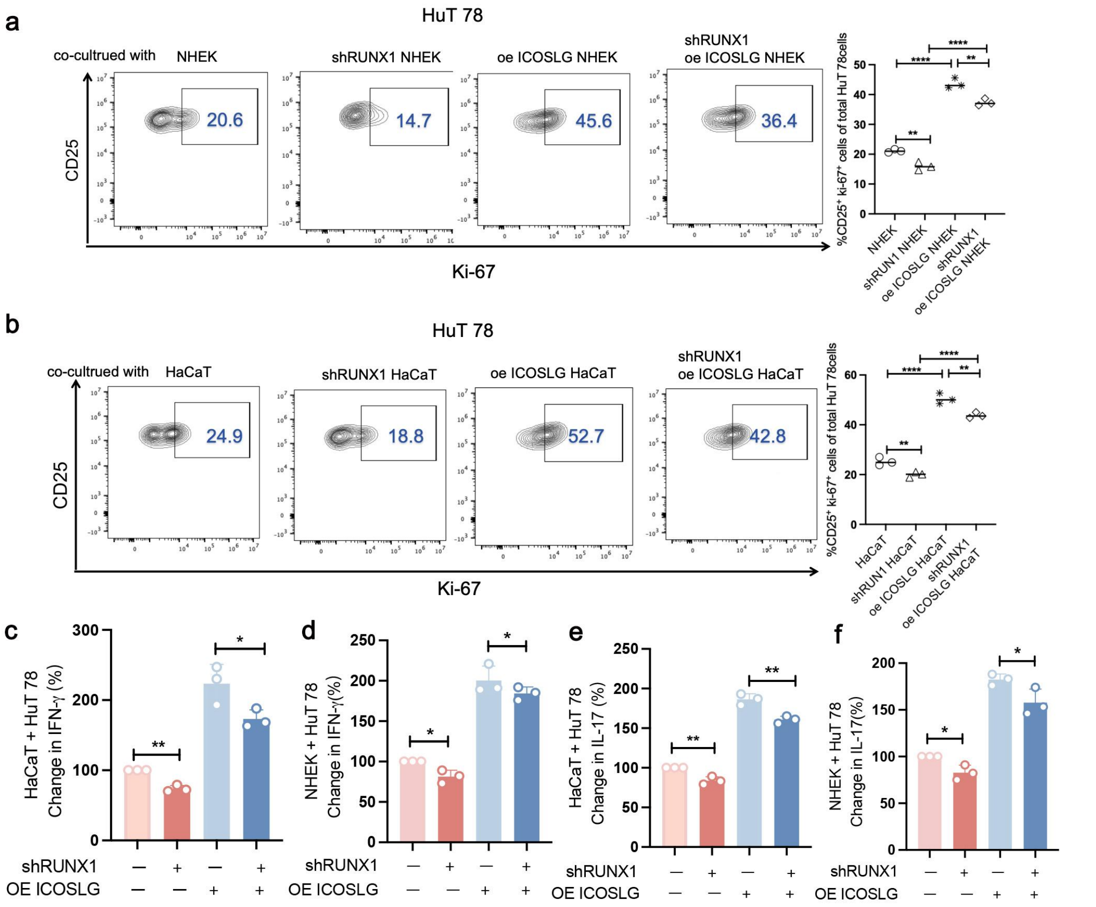

Supplementary Fig. 4 RUNX1 knockdown in keratinocytes decreased T cell secretion of pro-inflammatory cytokines.

(a-b) Flow cytometric analysis of HuT 78 cells using the following antibody panels: anti-CD25/Ki-67; (c-f) ELISA analysis of IFN-γ and IL-17 level in co-culture supernatant from RUNX1 knockdown and/or ICOSLG overexpression NHEK and HaCaT cells co-cultured with HuT 78 cells. Data are mean ± SD (n=3 independent experiments). One-way ANOVA with Tukey correction; **P* < 0.05, ***P* < 0.01, ****P* < 0.001, *****P* < 0.0001, ns (not significant).

Supplementary Fig. 5

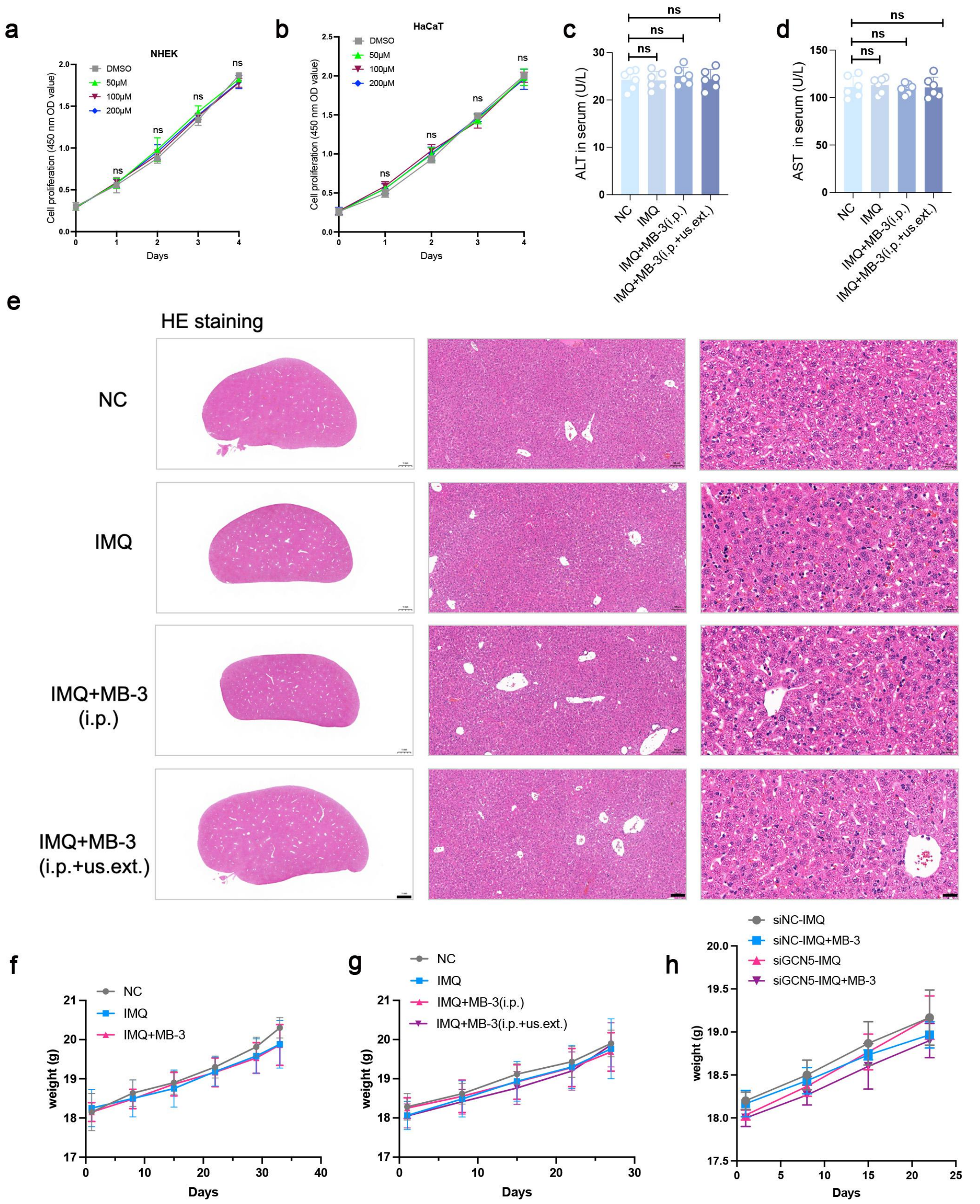

Supplementary Fig. 5 MB-3 exhibits a favorable safety profile with no detected effects on cell proliferation, hepatotoxicity, or mouse growth.

(a-b) Cell proliferation of NHEK and HaCaT cells treated with 50 μM, 100 μM, and 200 μM MB-3 was evaluated using a Cell Counting Kit-8 (CCK-8) assay. (c-d) ELISA analysis was performed to quantify serum alanine aminotransferase (ALT) and aspartate aminotransferase (AST) levels in mice. (e) H&E Staining of liver sections from the indicated groups. Scale bars, 1mm (left), 100 μm (middle), 25 μm (right). (f-h) Body weight was monitored in mice from study initiation until endpoint euthanasia for the indicated animal experiments. Data were showed as mean ± SD of three independent experiments and were analyzed by one-way ANOVA. ns represent no statistical significance. Graphs were drawn by GraphPad Prism 10.

Supplementary Fig. 6

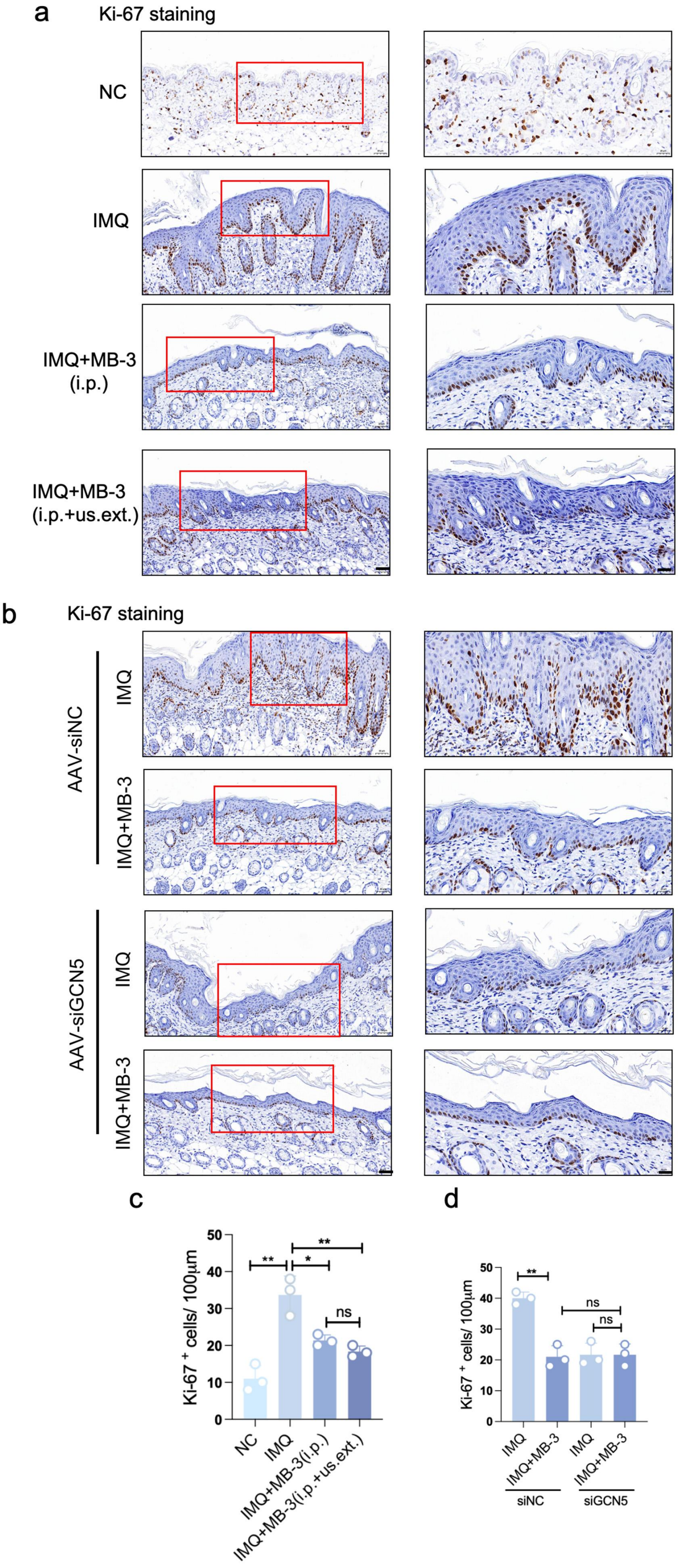

Supplementary Fig. 6 MB-3 inhibits the proliferation of keratinocytes.

(a-b) Immunohistochemical analysis of Ki-67 Expression for the indicated groups. Scale bars, 50 µm (left), 25 µm (right). (c-d) Ki-67^{+} cells per 100 µm area along the basement membrane (n = 3 areas). Data were showed as mean±SD of three independent experiments and were analyzed by one-way ANOVA with Tukey correction, and the indicated *P* value were shown.
